# Osteoid Osteoma of the Maxilla Presenting as Dental Implant Pain

**DOI:** 10.1155/2020/2092940

**Published:** 2020-07-23

**Authors:** R. Al Sadhan, A. Alosaimi, R. Al Shagroud, M. U. Zaman, M. S. Allahyani

**Affiliations:** ^1^Department of Oral Medicine and Diagnostic Sciences, College of Dentistry, King Saud University, Riyadh, Saudi Arabia; ^2^Dental University Hospital, King Saud University Medical City, King Saud University, Riyadh, Saudi Arabia; ^3^Department of Oral and Maxillofacial Surgery, Al Noor Hospital, Makkah, Saudi Arabia

## Abstract

Osteoid osteoma (OO) is a benign osteogenic lesion, regularly noticed in young individuals. A solitary lesion most frequently appears in long bones but is extremely rare in jawbones. Pain is a distinguishing characteristic of this lesion. Herein, we report a rare case of an OO in the right maxilla of a 37-year-old male presenting as pain associated with dental implants. Clinical and radiographic features were indicative of a benign neoplasia of boney origin. An excisional biopsy and histological examination of the lesion confirmed the diagnosis of osteoid osteoma. Surgical excision was followed by immediate relief of most of the pain. His follow-up visits were documented; complete relief of symptoms with no complications was observed during the postoperative period. There was no evidence of recurrence at a two-year follow-up. Osteoid osteoma of the maxilla may present as pain related to dental implants, and careful radiographic assessment of the entire jawbone should be considered if diagnosis of dental implant pain is unclear.

## 1. Introduction

Solitary osteoid osteoma (OO) is a rare benign osteogenic tumor of unknown etiology. First described in 1930 by Bergstrand and later classified by Jaffe in1935 [1], it was characterized as an offbeat clinical entity [2]. Walia et al. defined OO as “a small, oval, or roundish tumor-like nidus composed of osteoid and trabeculae of newly formed bone deposited within a substratum of highly vascularized osteogenic connective tissue” [3, 4]. It is characterized by its small size and severe, predominantly nocturnal, localized pain which is frequently relieved by the use of nonsteroidal anti-inflammatory drugs (NSAIDs). The pain may occur with both initial and recurrent disease [5]. Even though the true nature of this lesion is still unknown, different reports suggest it usually occurs in young adults under 30 years of age [6]. An OO is smaller in size than an osteoblastoma, with a central nidus that is usually less than 1 cm in diameter. The osteoid osteoma is more common than osteoblastoma and elucidates approximately 10% to 12% of all primary bone tumors [7–9]. This type of lesion accounts for 3% of all primary bone tumors and 10% of benign bone tumors. It tends to arise more in the long bones of the lower extremities than the long bones of the upper extremities. It may also involve the axial skeleton. According to Dorfman and Czerniak, it barely involves the craniofacial bones [7]. OO most commonly exhibits a prediction for long bones. It rarely occurs within the jaws, with the mandible more commonly affected than the maxilla [10, 11]. Multiple osteomas of the jawbones are seen in Gardner syndrome [12].

Since the original descriptions of osteoid osteoma were first published, 31 examples of isolated osteoid osteoma arising in the jawbones have been reported in the English-language literature until September 2019 [2, 4, 10, 13–40]. OO is seen in less than 1% of jawbones [2]. But it has hardly been described in the jaws [6]. Knowing the proper diagnosis and treatment plan are essential for clinicians because of the specialty and rareness of this tumor.

Hence, to acquaint our awareness in the concerned field, such case reports should be discussed. However, its occurrence in the anterior wall of the maxillary sinus wall just below the infraorbital rim is a rare entity. Here, we presented a rare case report of OO of the maxilla in a 37-year-old patient presenting as pain associated with dental implants. This case report highlights that osteoid osteoma of the maxilla may present as pain related to dental implants and careful radiographic assessment of the entire jawbone should be considered if diagnosis of dental implant pain is unclear.

## 2. Case Report

A 37-year-old male patient was seen at the Oral and Maxillofacial Clinic of the Dental University Hospital, King Saud University, Riyadh, Saudi Arabia. He was complaining of localized severe pain in the right maxilla. No swelling or tenderness was noted clinically. The patient had previously undergone the replacement of missing maxillary premolars and molars on the same side with dental implants, and thus, implant-related complication was suspected although the implants were loaded and functional with intact periodontium.

## 3. Radiographic Examination

**Figure 1:**
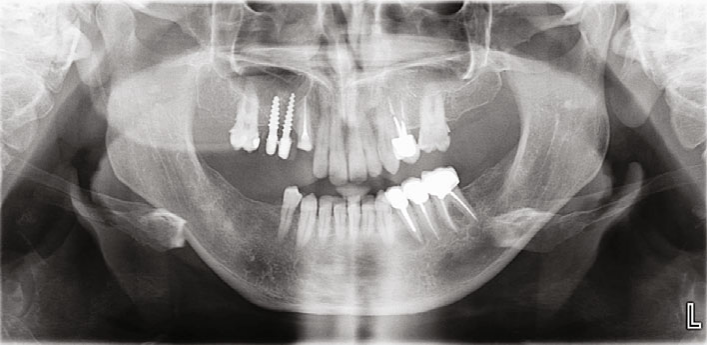
Initial panoramic radiograph.

**Figure 2:**
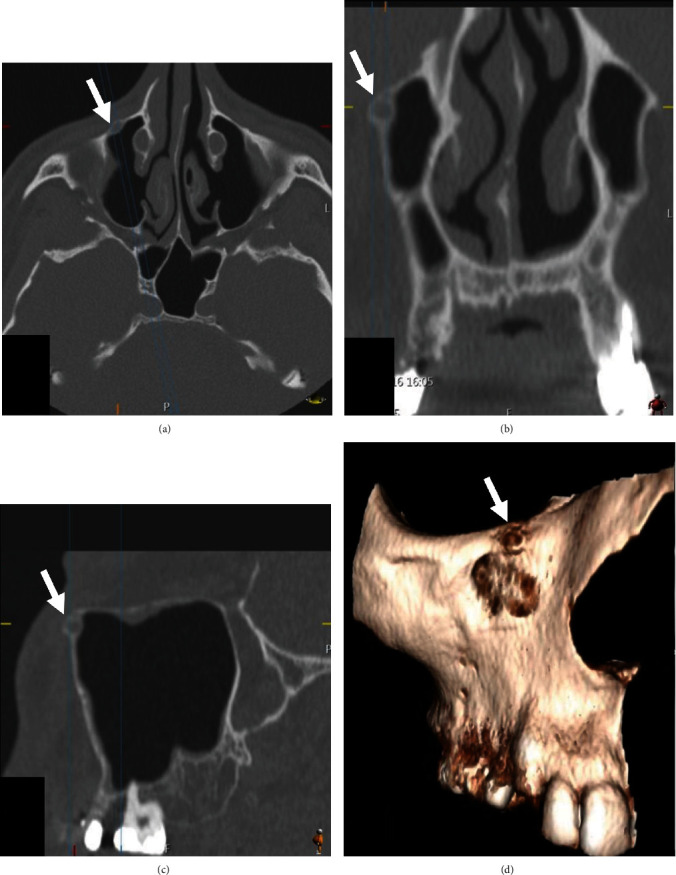
CT examination of the right maxilla in the bone window: (a) axial section, (b) corrected sagittal section, (c) coronal section, and (d) surface rendering; white arrows showing a small, well-defined, corticated, spherical, radiolucency in the superioanterior wall of the right maxillary sinus wall just below the infraorbital rim and medial to the infraorbital foramen.

A panoramic radiograph (Figure 1) and CT examination (Figure 2) were requested to assess the area, and the images showed a severe marginal alveolar bone loss in the area of the missing upper right posterior teeth and inferior extension of the maxillary sinus floor. The two previously placed dental implants in the area of the missing maxillary right premolars and molars were covered with a very thin surrounding residual alveolar ridge, and localized thickening of the adjacent maxillary sinus lining was noted. Maxillary sinus mucositis of odontogenic origin was initially suspected to be the cause of the patient's pain. But this finding was not fully consistent with the chief complaint of the patient who described his localized pain by pointing at his face just below his right orbital rim and reported that he had severe nocturnal pain at this spot. When the patient signs and symptoms were correlated with the CT examination, a small (3.5 mm in diameter), well-defined, corticated, spherical radiolucency in the superior anterior wall of the right maxillary sinus wall just below the infraorbital rim and medial to the infraorbital foramen was noted. The lesion caused minimal expansion of the boney boundaries especially anteriorly and posteriorly. The CT numbers (HU) indicated that the content is similar to soft tissue. The radiographic observation of a small, well-defined, corticated, unilocular, minimally expansile radiolucency that appears to have originated inside the bone was suggestive of a benign neoplasia of boney origin. The primary radiographic differential diagnosis for small benign neoplasia of the bone that causes pain is osteoid osteoma. Based on the case history, clinical findings, and radiographic presentation, an excisional biopsy of the lesion was planned.

## 4. Surgical Technique

**Figure 3:**
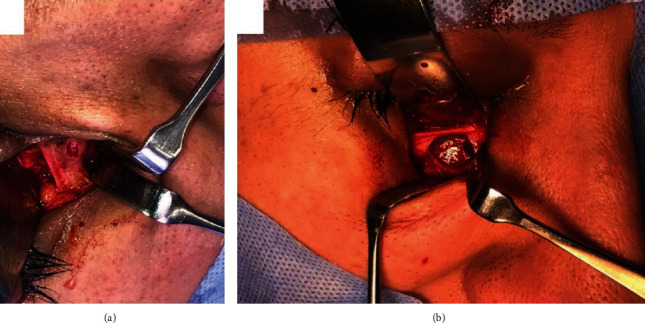
Intraoperative photographs showing (a) the lesion on the right inferior orbital rim and medial to infraorbital foramen and (b) after complete removal of the lesion with preserving inferior orbital rim.

**Figure 4:**
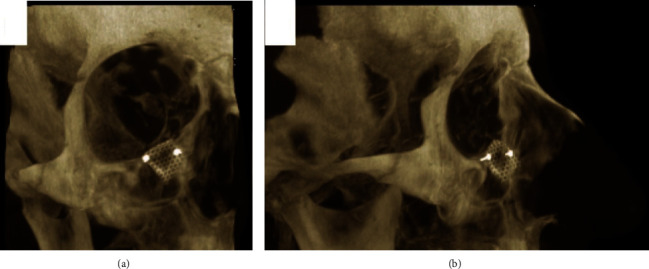
Thick-slice CT sections in the maximum intensity projection (MIP) in (a) frontal and (b) lateral views showing the reconstruction mesh in place.

On the day of the surgery, the patient was brought to the operating theater where the surgical intervention under general anesthesia in a sterile manner was done; the lesion was approached via a transcutaneous incision of the right lower eyelid (midlid incision) after protecting the right eye by employing temporary suture tarsorrhaphy. Sharp and blunt dissection was done exposing the inferior orbital rim adequately. A small bony swelling was located on the facial aspect of the medial portion of the right inferior orbital rim just medial to the right infraorbital nerve and foramen with no violation of the foramen. The lesion was a nodule-like bony mass with central crater-like defect, brownish-red, and gritty. It measured about 3 to 4 mm in dimension and was distinct from the surrounding normal bone (Figure 3(a)). The lesion was excised completely by using a straight surgical bur and chisels. The right infraorbital nerve was protected and not violated (Figure 3(b)). The residual bony defect was reconstructed by using 1 mm thick titanium mesh (Figure 4). The surgical site was irrigated thoroughly with normal saline; the surgical wound was sutured back in layers by using absorbable suture and the lid skin by 6/0 proline suture.

## 5. Microscopic Findings

**Figure 5:**
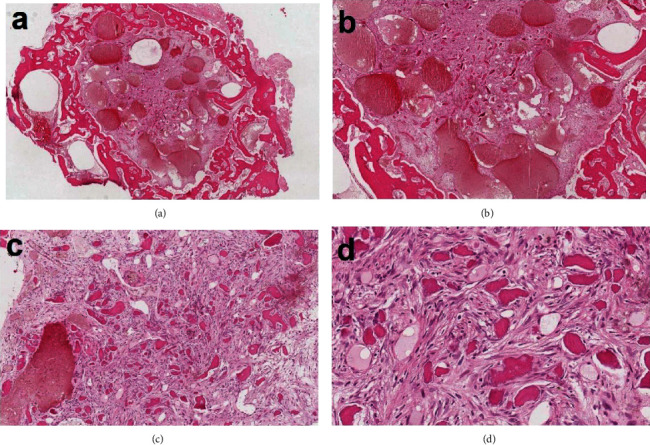
H&E-stained histopathologic slides of the excised specimen showing (a) a well-circumscribed and highly vascular tumor surrounded by a thick ring of mature bone. (b) The tumor composed of dilated blood vessels, area of hemorrhage, and numerous scattered irregular calcifications embedded in a fibrous stroma. (c) Cellular fibrous connective tissue containing irregular bony trabeculae. Area of hemorrhage is present. (d) The bony trabeculae are acellular, and the stroma contains plumped osteoblasts with ample cytoplasm and hyperchromatic nuclei.

**Figure 6:**
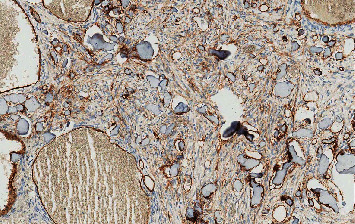
CD34 immunoreactivity in the endothelial lining of the blood vessels.

The histopathologic examination of the excised specimen (Figure 5) revealed a well-circumscribed, round, hemorrhagic benign bone-forming tumor surrounded by a thick ring of the mature bone. The tumor was composed of haphazard, irregular trabeculae of woven bone rimmed by osteoblast and embedded in a loose fibrous stroma. The stroma contains dilated blood vessels and occasional hemorrhage. Few scattered osteoclasts are seen often on the surface of the trabeculae. Endothelial cell marker antibodies CD31 and CD34 were used to highlight the wall of the blood vessels (Figure 6). Histopathologically, osteoblastoma and osteoid osteoma can look very similar, and the distension depends on the size of the lesion. Therefore, the clinical, radiographic, and histopathological correlations make us favor osteoid osteoma as a final diagnosis.

## 6. Postoperative Patient Course

From day one postoperatively, the patient felt a great improvement in the pain intensity which became mild pain and discomfort (about 3 to 4 out of 10) according to his visual analog scale. His follow-up visits were documented regarding his pain perception including the nocturnal pain, which revealed that he is almost in pain-free status. His surgical site after two years healed nicely without noticeable scarring.

## 7. Discussion

Osteoid osteomas are benign skeletal tumors that are portrayed by an intracortical nidus with a variable amount of calcification, sclerosis, and bone marrow edema [1]. Jaffe described OO as a benign neoplasm with an inflammatory process and considered it a variant of osteoblastoma [1], occurring usually in young adults, with pain as a dominant feature. The pathogenesis of osteoid osteoma has remained controversial. Some authors consider it a neoplasm while some believe it is mainly an inflammatory process. The precise nature is still unclear [14]. Some authors consider it a slow-growing dormant neoplasm or an inflammatory reaction or aftereffect of an unusual healing process [41]. Regarding the nature and genesis of OO, numerous theories have been proposed [1]. The growth of OO is limited, and the lesion size is generally less than 2 cm. A solitary type of this tumor is extremely rare in the head and neck region but relatively common in long bones of the lower limbs [29]. OO typically involves the tibia, femur, fibula, humerus, and vertebral arch [42]. Jaw involvement is rare, with the lingual surface and lower border of the body of the mandible being the most common sites [42]. Its occurrence in the maxillary sinus wall just below the infraorbital rim is rarer.

Clinically, osteoid osteomas are characterized by dull, throbbing, intermittent, local, and nocturnal pain relieved with nonsteroidal anti-inflammatory drugs such as aspirin and are associated with slight local swelling [36]. For decoding the reason for pain, diverse theories were mentioned. Jaffe outlined pain seen in osteoid osteoma as being attributable to the arteriolar blood supply of the lesion [1]. In the current case, the pain was severe in nature, subsiding significantly after the surgery. It is seen to be more common in males compared to females with a ratio of 2 : 1, commonly affecting in the second and third decades of life, and is rarely seen in individuals over 30 years of age [42, 43]. However, the patient reported was a male who was 37 years.

Radiographically, OO is a small, radiolucent intracortical nidus, less than 1 cm in diameter, surrounded by a large, dense sclerotic zone of cortical thickening [44]. Jaffe pointed out that the radiographic features of osteoid osteoma were most important in the ultimate diagnosis of the lesion. He mentioned that the nidus was more radiolucent than radiopaque and that it was surrounded by a reactive radiopacity that extended a variable distance from the nidus [42]. The radiolucent nidus is an ominous sign of a fully mature OO whereas the radiopaque nidus suggests a less mature lesion. Radiolucency with central calcification, located in the cortical bone with surrounding sclerotic bone, is the characteristic feature of osteoid osteoma [3]. The present case was observed in the maxillary anterior region, which is quite uncommon comparing with previously cited cases [2, 4, 10, 13–40]. However, in dentistry, such cases could be underdiagnosed since such a small lesion can be missed on a panoramic radiograph because of the complex anatomic site of the jaws, and CT or CBCT is a useful imaging modality for this diagnosis [3, 30].

The histopathological examination of OO differs according to the site and age of the lesion. Huvos illustrated three specific evolutionary stages of modification of OO. Originally, dense osteoblasts are seen proliferating actively in a highly vascularized stroma followed by deposition of the osteoid matrix between the osteoblasts in the intermediate phase. In the mature stage, the osteoid is transformed into well-calcified, compact trabeculae of atypical bone, which is neither typically woven nor lamellar [2]. Sometimes OO showed possible histological patterns of a neoplastic lesion in the later stages of development [3]. An inflammatory lesion illustrated pain as its constant feature [14]. The cases previously published involving the jaws were abstracted as a type of nidus, more brittle in nature, composed of osteoid tissue predominantly. Describing the microscopic study, Chaudhary and Kulkarni mentioned that a broken nidus may be misguided as granulation tissue and the older lesion might show atypical bone modeled from sheets of osteoid trabeculae [42].

Previously reported cases mentioned several lesions as a differential diagnosis of OO, such as ossifying fibroma, peripheral osteoma, osteoblastoma, osteosarcoma, and fibroosseous lesions. Ossifying fibroma and peripheral osteoma are usually asymptomatic, increase in size, lack nidus, and cause resorption and displacement of teeth [45]. Ossifying fibroma (OF) may have similar radiographic features, presenting as circumscribed well-demarcated bone swellings but lack the presence of a central nidus. OF is typically asymptomatic; therefore, if nocturnal pain is a presenting feature, it can also aid differentiation between these two lesions [43]. Because of clinical, radiographic, and histologic similarities between osteoid osteoma and osteoblastoma, it is very difficult to achieve a differential radiographic diagnosis between them. Also, OO is more painful than osteoblastoma, but this criterion can be subjective, and thus, the patient must provide a clear history of pain [35]. Aspirin and other nonsteroidal anti-inflammatory drugs are very useful in pain associated with OO, but not for an osteoblastoma [45]. Diagnosis of OO is made when the presence of a radiopaque nidus surrounded by sclerotic new bone formation is noted [35]. OOs are defined by their limited growth and size of less than 2 cm in diameter, distinguishing them from osteoblastoma which is usually greater than 2 cm in size, with most 3–10 cm [43]. Osteosarcoma can be excluded based on the clinical behavior of the lesion, which would present as a more rapidly growing painful swelling as well as different histopathological presentation. Fibrous dysplasia can be differentiated from OO radiographically, as fibrous dysplasia is poorly defined with a “ground-glass” appearance. Histologically, fibrous dysplasia shows features typical of any fibrous osseous lesion, with the replacement of normal bone by a variably cellular stroma within which abnormal bone is noted; however, unlike OO, fibrous dysplasia shows little or no osteoblastic rimming [43].

In the present case, the diagnosis of osteoid osteoma was made considering the reported symptom of gradually increasing pain (with which the patient presented to the emergency dental clinic), the CT radiographic appearance, and the histopathologic examination of the specimen.

Studies suggested complete excision should be the treatment of choice with intact removal of the nidus. Recurrence of the lesion depends upon the complete removal of the lesion. To date, no history of malignant transformation of OO has been reported [3, 42]. Albeit some reports of spontaneous remission of osteoid osteoma, complete excision of the osteoid osteoma nidus is the ultimate treatment of choice as it brings immediate relief of pain and completely cures the patient [10].

Wexell et al. recently reported a 7-year follow-up of a 37-year-old woman with Gardner syndrome who had multiple osteomas in both jaws with dental implant treatment with no complication. However, in that case, the patient had known long standing preexisting nonpainful multiple osteomas as part of the Gardner syndrome [46].

## 8. Conclusion

Herein, we reported a case of osteoid osteoma that occurred in the right maxilla of an adult male patient. The correct diagnosis was achieved by considering clinical, radiographic, and pathological features. To avoid confusion with similar bony pathosis, the treating physician should have a clear concept and keen observation skill. Osteoid osteoma is a benign bone lesion that occurs very rarely in the jawbones. Due to the rarity of this lesion, it is essential to report these cases to increase awareness among dental surgeons worldwide. Osteoid osteoma of the maxilla may present as pain related to dental implants and careful radiographic assessment by CT or CBCT of the entire jawbone should be considered if diagnosis of dental implant pain is unclear.

## Data Availability

There is no underlying data supporting this case report.
